# *Ficus palmata* Forskål (beles adgi) as a source of milk clotting agent: a preliminary research

**DOI:** 10.1186/s13104-020-05293-x

**Published:** 2020-09-18

**Authors:** Desta Berhe Sbhatu, Hailekiros Tadesse Tekle, Kiros Haddish Tesfamariam

**Affiliations:** 1 Department of Biological and Chemical Engineering, Mekelle Institute of Technology, Mekelle University, PO Box 1632, Mekelle, Ethiopia; 2 Department of Forensic Medicine, School of Medicine, College of Health Sciences, Mekelle University, PO Box 231, Mekelle, Ethiopia

**Keywords:** Coagulation, Ficin, *Ficus palmata* Forskål, Milk, Powder

## Abstract

**Objective:**

The demand for cheese, the insufficient supply and high cost of rennet, and the ethical issues of harvesting rennet oblige us to search for suitable alternatives of finding new proteases from plants. *Ficus palmata* Forskål (Moraceae) is one of the plants producing a protease called ficin that coagulates fresh milk. This study aims to study the milk coagulating abilities of bark, leaf, and stem powders of *F. palmata* Forskål.

**Results:**

Stem powder has yielded better results. Chemical analyses of the powders have revealed that the percentage of crude protein of leaf, bark, and stem powders were 4.17, 7.39, and 16.26. This is an indication of the suitability of stem biomass as source of the enzyme of interest. Further research needs to aim at qualitative and quantitative analyses of milk-coagulating enzymes of *F. palmata* Forskål stem biomass to get new insights into industrial extraction of the enzymes of interest.

## Introduction

Cheese is an important dairy product of high economic and nutritional significance. Cheese preserves essential nutrients in milk and is an excellent source of proteins, fats, minerals, and vitamins. Cheese making is carried out using animal rennin—called rennet. Rennet helps in coagulating the casein of milk. It is characterized by low proteolytic and high milk clotting activity [1]. The milk clotting property of an enzyme is important with regard to the quality and yield of the cheese [2, 3]. The growing demand for cheese, the insufficient supply and high cost of rennet, and the associated ethical issues of harvesting rennet oblige us to search for a suitable alternative. There is a growing research interest in recent years towards finding new proteases with milk coagulation potential from plants [4–6]. This study aims to conduct a preliminary research on milk coagulating capacity of bark, leaf and stem powders of *Ficus palmata* Forskål (Moraceae).

*Ficus palmata* belongs to genus *Ficus* that includes about 750 tree and shrub species, with several medicinal plants, primarily occurring in subtropical and tropical regions of the world. *Ficus* is remarkable for the large variation in the habits of its species [7]. *Ficus* species are among the first plants to be cultivated by humans [8]. The plants are usually monoecious with small flowers without petals and nectarines [9]. The fruits of *Ficus* species vary from yellowish-green to coppery, bronze, or dark-purple. They are known for their nutritive value, consumed as fresh or dry and for their mild laxative activity and high alkalinity [10]. They are propagated through cutting of mature woods or grafts [11].

## Main text

### Materials and methods

#### Identification and collection of plant materials

*F. palmata* Forskål is the most common fig in Ethiopia. It is shrub to small tree growing between 1700 and 2400 masl in water courses or river banks. It releases white latex up on removing its unripe fruits, breaking its leaf petioles, cutting its shoot tips, and slashing its stem [12]. Whereas the fruits are eaten, the latex is used in treating skin warts [12, 13]. Bark, leaf and stem biomass was collected by the researchers from wild stands in *Ab’ala* and *Hiwane*, Northeastern Ethiopia following ethical and legal procedures. Collected specimens include barks (green and fibrous), leaves (green to deep green), and stem (woody). The specimen is identified by Professor Mirutse Giday. Specimens of the plant are deposited in the Aklilu Lemma Institute of Pathobiology, Addis Ababa University (Voucher No. of AS-16-2017).

#### *Preparation of F. palmata leaf, bark and stem powders*

The bark and leaf biomass of the plant was dried in a ventilated oven at 45 °C for 24 h; and immediately milled finely into powder using grinding machine. Stem biomass was dried at room temperature for 4 days and ground into powder. The powdered biomasses of bark and leaf were treated with methanol to extract the chlorophyll. Chlorophyll extraction was carried out by treating 100 g of the biomass with 80 mL methanol (purity grade 98%) in Erlenmeyer flasks. Flasks holding the mixture were stirred every 30 min to enhance the extraction. Then, the flasks were placed in a Sonicator Bath (Branson 8210) and sonicated at 40 °C for 30 min for further stirring and mixing. The mixture in each flask was filtered using filter paper; and the flasks were washed with 30 mL and then with 50 mL ethanol. Filtrates were poured into round-bottom flasks and the solvents were concentrated in vacuo at about 11 mm Hg up to 5–10 mL using rotavapor with the help of water bath at 40 °C. Finally, the biomass was put in 30 mL vessels to evaporate the solvent and left open overnight in a well-ventilated hood to further evaporate traces of the solvent. About 150 g of biomass was collected for each plant part. On the other hand, powdered biomass of stem was mixed with distilled water (250 g/L, v/w) in Erlenmeyer flask, and was shaken for 15–20 min. Each mixture was filtered using Whatman No. 1 filter paper. The biomass was, then, collected and centrifuged at 4000× for 10 min to reduce the fibers. Finally, the precipitates were loosened, put into distilled water, and filtered with the same filter paper. These procedures helped us harvest 150 g of powdered biomass.

#### Milk coagulation using crude biomass powders

The coagulation abilities of bark, leaf, and stem powders were tested with ½, 1, 1½, and 2 teaspoons for 50, 100, 150, 200, 250, 300, 350, 400, 450, and 500 mL of fresh cow milk. This test was made to identify the minimum effective amount of *F. palmata* that can coagulate 50 mL of fresh cow milk. Data on coagulating abilities of the powders were expressed in terms of time of coagulation, hence time of coagulation of the treatments were recorded. Up on establishing the minimum effective amount of powder (i.e. ½ teaspoons) to coagulate 50 mL fresh cow milk, the coagulating capacities of bark, leaf, and stem powders were tested with 30 replications each. Data on time for coagulation (in hours) were collected.

#### Comparison of coagulating capacities of stem, bark, and leaf extracts

Once it was established that ½ teaspoon of powder of bark, leaf, and stem was sufficient to coagulate 50 mL fresh cow milk, their coagulating capacities—in terms of coagulation time—were compared. Powder extracted from bark, leaf, and stem biomass was mixed with fresh cow milk at the rate of ½ teaspoon per 50 mL of milk. There were 30 treatments for each of the plant powders. Data on coagulation time were collected and organized for analysis.

#### Gross chemical analyses of bark, leaf, and stem powders

Bark, leaf, and stem biomass of *F. palmata* was air-dried in shade for 72 h. The dried biomass was grinded grossly with mortar and pestle, and finely with grinding machine to collect enough powder. Samples of powders were then sent for gross chemical analyses in JIJE LABOGLASS Pvt. Ltd. Co., Addis Ababa, Ethiopia. The analyses were made for moisture content (AOAC Official Method 925.10), crude fat (AOAC Official Method 920.39—Soxhlet/Gravimetric), crude protein (ES ISO 1871:2013), crude ash (AOAC Official Method 923.03), carbohydrate (by difference), and crude fiber (AOAC Official Method 962.09—Gravimetric).

#### Statistical analyses

Collected data were analyzed and mean values were compared using appropriate inferential statistical methods at a priori established significance level of *p* ≤ 0.01.

### Results

#### Establishing the optimum amount powder for effective coagulation

The coagulating capacity of stem powder increases with increasing the amount of powder used. A ½, 1, and 1½ teaspoons of stem powder were ineffective in initiating any coagulation of cow milk in 200, 300, and 400 mL, respectively. Similar tests were carried out with bark and leaf powders. In both cases, the coagulating capacities of the powders increase with increasing their amounts and decreases with increasing the volume of milk. Thus, ½, 1, and 1½ teaspoons of bark powders were ineffective in coagulating cow milk in 150, 250, and 300 mL, respectively. Likewise, ½, 1, and 1½ teaspoons of leaf powder were ineffective in causing the coagulation of cow milk in 150, 250, and 400 mL, respectively (Table 1). In addition to these observations, Table 1 shows that the same amount of powder from bark, leaf, and stem have varied in their coagulating capacity of the same volume of milk.

**Table 1:** Coagulating capacity of bark, leaf, and stem powders of *F. palmata*

Milk volume (mL)	Coagulating time (in h) of *F. palmata* powder in teaspoons											
	Bark				Leaf				Stem			
	½	1	1 ½	2	½	1	1 ½	2	½	1	1 ½	2
50	0.91	0.78	0.60	0.46	1.33	1.03	0.81	0.63	0.70	0.50	0.32	0.21
100	1.30	1.01	0.76	0.65	1.83	1.33	1.01	0.75	1.40	1.20	0.75	0.45
150	‒	1.25	1.06	0.85	‒	1.58	1.26	0.95	1.70	1.42	0.91	0.50
200	‒	1.45	1.20	1.05	‒	2.25	1.55	1.20	‒	1.58	1.30	0.91
250	‒	‒	1.58	1.26	‒	‒	1.83	1.48	‒	1.75	1.53	1.16
300	‒	‒	2.00	1.45	‒	‒	2.36	1.65	‒	‒	1.83	1.41
350	‒	‒	‒	2.41	‒	‒	2.83	2.20	‒	‒	2.11	1.63
400	‒	‒	‒	2.96	‒	‒	‒	3.25	‒	‒	‒	2.17
450	‒	‒	‒	3.33	‒	‒	‒	4.13	‒	‒	‒	2.58
500	‒	‒	‒	3.80	‒	‒	‒	5.01	‒	‒	‒	3.01

#### Coagulating capacities of stem, bark, and leaf extracts

This experiment showed that the average time required to coagulate 50 mL of milk with ½ teaspoon stem powder (43.63 ± 1.16 min) is significantly lower than bark powder (56.70 ± 1.37 min) (F = 1592.66; p ≤ 0.000) and leaf powder (78.73 ± 1.28 min) (F = 12,343.75; p ≤ 0.000). Likewise, the average time required to coagulate 50 mL of milk with ½ teaspoon of bark powder is significantly lower than with leaf powder (F = 4134.00; p ≤ 0.000) (Table 2).

**Table 2:** Coagulating capacity of ½ teaspoon of bark, leaf, and stem powder for 50 mL milk

Sources of powder	Mean time (SD) in min		ANOVA		
	Mean 1	Mean 2	*df*	F	*p*
Stem vs. leaf	43.63 (1.16)	78.73 (1.28)	59	12,343.75	0.000
Stem vs. bark	43.63 (1.16)	56.70 (1.37)	59	1592.66	0.000
Bark vs. leaf	56.70 (1.37)	78.73 (1.28)	59	4131.00	0.000

#### Gross chemical analyses of bark, leaf, and stem powders

Findings of the chemical analyses are given in Table 3. Stem powder has more than twice and nearly four times crude protein compared to bark and leaf powders, respectively. This may be the reason for the better coagulating capacity of stem powder.

**Table 3:** Gross chemical analyses of bark, leaf, and stem powder

S. no	Testing parameters	Source of dry powder (%)		
		Bark	Leaf	Stem
1	Moisture content	8.62	6.64	7.64
2	Crude ash	10.58	3.11	15.74
3	Crude protein	7.39	4.17	16.26
4	Crude fat	6.02	2.43	5.63
5	Carbohydrate	67.39	83.66	54.74
6	Crude fiber	24.80	50.10	12.90

### Discussion

Plant extracts are often used for home-based cheese making and instant on-farm yogurt making in many communities. Many studies reported that many plants are sources of various proteases used in cheese making including papain, bromelin, ficin, oryzasin, and cucumisin. *Solanum dubium* and other *Solanum* species [4, 14–16], Dead Sea apple (*Calotropis procera*) [17], *Moringa oleifera* [18], *Balanites aegyptiaca* fruit pulp [19], *Cynara scolymus* L. flower extracts [20–23], *Cynara cardunculus* L. [24], *Albizia julibrissin* young seeds [25], sunflower and *Albizia* seeds [26], melon fruit extracts [27], and *Lactuca sativa* leaf extracts [28] are sources of cheese making proteases. Ethiopian lowlands and highlands are home to pastoralist and agrarian communities. As the floristic compositions of the many agro-climatic zones of Ethiopia are fairly distinct, the diverse pastoralist and agrarian communities use different plants to coagulate milk at home and on the farm. Typical examples are the use of *F. palmata* latex for coagulating fresh goat milk in many parts of Northern Ethiopia; the use of fresh leaf of *Ocimum lamiifolium* Hochst. ex Benth. for coagulating fresh cow and goat milk in many parts of Eastern Ethiopia, and use of *Solanum* fruit juice in coagulating cow and goat milk in Northeastern Ethiopia.

### Conclusion

The study showed that two teaspoons of plant powder can coagulate 500 mL fresh cow milk in 3 to 5 h and samples of the stem powder have a high percentage of crude protein. Treatment of barks and leaves with methanol removes chlorophyll leading to the reduction of crude protein content. However, since chlorophyll does not involve in coagulating milk, it can be claimed that the crude protein in the powders is the source of milk-coagulating enzymes. Hence, this study has established that *F. palmata* biomass is an excellent source of milk-coagulating enzymes. Further studies need to aim at qualitative and quantitative biomolecular analyses of milk-coagulating enzymes of the plant.

## Limitations

The test aiming at establishing the optimum amount of powder for effective coagulation was not replicated because it requires large volume of milk.

## Data Availability

The datasets used and/or analyzed during the current study are available from the corresponding author on reasonable request. Specimens of the plant have been deposited in the Aklilu Lemma Institute of Pathobiology, Addis Ababa University (Voucher No. of AS-16-2017).

## Abbreviations

AOAC: Association of Official Analytical Chemists
msal: Meters above sea level
SD: Standard deviation
v/w: Volume by weight
